# Disruption of *SF3B1* results in deregulated expression and splicing of key genes and pathways in myelodysplastic syndrome hematopoietic stem and progenitor cells

**DOI:** 10.1038/leu.2014.331

**Published:** 2014-12-23

**Authors:** H Dolatshad, A Pellagatti, M Fernandez-Mercado, B H Yip, L Malcovati, M Attwood, B Przychodzen, N Sahgal, A A Kanapin, H Lockstone, L Scifo, P Vandenberghe, E Papaemmanuil, C W J Smith, P J Campbell, S Ogawa, J P Maciejewski, M Cazzola, K I Savage, J Boultwood

**Affiliations:** 1 LLR Molecular Haematology Unit, NDCLS, RDM, University of Oxford, Oxford, UK; 2 Department of Hematology Oncology, Fondazione IRCCS Policlinico San Matteo, Pavia, Italy; 3 Department of Molecular Medicine and Medical Therapy, University of Pavia, Pavia, Italy; 4 Department of Translational Haematology and Oncology Research, Taussig Cancer Institute, Cleveland, OH, USA; 5 The Wellcome Trust Centre for Human Genetics, University of Oxford, Oxford, UK; 6 Department of Oncology, University of Oxford, Oxford, UK; 7 Center for Human Genetics, Katholieke Universiteit Leuven/University Hospital Leuven, Leuven, Belgium; 8 Wellcome Trust Sanger Institute, Wellcome Trust Genome Campus, Hinxton, UK; 9 Department of Biochemistry, Downing Site, University of Cambridge, Cambridge, UK; 10 Cancer Genomics Projects, Graduate School of Medicine, Tokyo, Japan; 11 Centre for Cancer Research and Cell Biology, Queen's University Belfast, Belfast, UK

## Abstract

The splicing factor *SF3B1* is the most commonly mutated gene in the myelodysplastic syndrome (MDS), particularly in patients with refractory anemia with ring sideroblasts (RARS). We investigated the functional effects of *SF3B1* disruption in myeloid cell lines: *SF3B1* knockdown resulted in growth inhibition, cell cycle arrest and impaired erythroid differentiation and deregulation of many genes and pathways, including cell cycle regulation and RNA processing. MDS is a disorder of the hematopoietic stem cell and we thus studied the transcriptome of CD34^+^ cells from MDS patients with *SF3B1* mutations using RNA sequencing. Genes significantly differentially expressed at the transcript and/or exon level in *SF3B1* mutant compared with wild-type cases include genes that are involved in MDS pathogenesis (*ASXL1* and *CBL*), iron homeostasis and mitochondrial metabolism (*ALAS2, ABCB7* and *SLC25A37*) and RNA splicing/processing (*PRPF8* and *HNRNPD*). Many genes regulated by a DNA damage-induced BRCA1–BCLAF1–SF3B1 protein complex showed differential expression/splicing in *SF3B1* mutant cases. This is the first study to determine the target genes of *SF3B1* mutation in MDS CD34^+^ cells. Our data indicate that SF3B1 has a critical role in MDS by affecting the expression and splicing of genes involved in specific cellular processes/pathways, many of which are relevant to the known RARS pathophysiology, suggesting a causal link.

## Introduction

The myelodysplastic syndromes (MDS) are a heterogeneous group of clonal hematopoietic stem cell (HSC) malignancies characterized by blood cell dysplasia and peripheral blood cytopenia. Approximately 30–40% of MDS patients will develop acute myeloid leukemia (AML).^1^

The recent discovery of somatic splicesomal mutations in MDS has revealed a new leukemogenic pathway involving spliceosomal dysfunction.^2, 3^ Somatic mutations in the splicing factor genes *SF3B1*, *U2AF1*, *SRSF2* and *ZRSR2* are frequent in MDS patients.^4^ Importantly, these genes encode proteins that are all involved in 3′-splice site recognition during pre-messenger RNA (pre-mRNA) processing. Splicing factor gene mutations occur in over 50% of MDS patients, are highly specific to this disorder, and occur in a mutually exclusive manner.^5, 6, 7^

*SF3B1*, encoding a core component of the U2 small nuclear ribonucleoprotein, involved in the recognition of the branchpoint sequence, is the most commonly mutated gene found in MDS (20–28% of all patients).^2, 8, 9^ We and others have shown that mutations of the splicing factor *SF3B1* are found in a high percentage (>70%) of MDS patients whose disease is characterised by the presence of ring sideroblasts, including both refractory anemia with ring sideroblasts (RARS) and refractory cytopenia with multilineage dysplasia and ring sideroblasts (RCMD-RS).^2, 8^ The close association between *SF3B1* mutation and the presence of ring sideroblasts is consistent with a causal relationship and makes this the first gene to be strongly associated with a specific morphological feature of MDS. Ring sideroblasts are characterised by an excess accumulation of iron in the mitochondria of erythroblasts,^10^ and *SF3B1* mutant RARS cases show altered iron distribution characterised by coarse iron deposits compared with wild-type RARS cases.^11^ *SF3B1* mutations are generally more prevalent in low-risk MDS and have been shown to be independent predictors of favorable clinical outcome in MDS in most studies.^8, 11^ The clinical consequences of *SF3B1* mutation in MDS are clear, but the functional consequences of these mutations in human cells remain poorly understood. Altered RNA splicing has been suggested as the mechanism underlying the observed phenotypic changes concomitant to splicing factor gene mutations, including *SF3B1*; ^3, 12, 13^ however, the target genes in the HSC of MDS cases with *SF3B1* mutations are yet to be defined.

*SF3B1* mutations in MDS are primarily heterozygous point mutations. The presence of hotspots and the absence of nonsense or frameshift mutations in *SF3B1* in MDS patients suggest that *SF3B1* mutations are likely to be gain/change-of-function (neomorphic) mutations. A heterozygous *Sf3b1*^+/−^ knockout mouse model has been shown to develop ring sideroblasts, suggesting that haploinsufficiency of *SF3B1* may lead to their formation.^11^ Recent similar studies have not made this observation, however.^14, 15^ Thus, it is yet to be determined whether *SF3B1* mutations found in MDS are loss-of-function mutations or gain/change-of-function mutations. In this study, we thus evaluated the effects of *SF3B1* knockdown on cell growth, gene expression and splicing in a range of myeloid cell lines and performed RNA sequencing (RNA-Seq) on the CD34^+^ cells of MDS patients harboring *SF3B1* mutations.

## Materials and methods

### Myeloid cell lines culture

K562, HEL, TF1 and SKM1 cells were cultured in Roswell Park Memorial Institute medium 1640 (Sigma-Aldrich, Gillingham, UK) containing 10% fetal bovine serum, at 37 °C and 5% CO_2_. TF1 and SKM1 cultures were supplemented with 2 and 1 ng/ml of granulocyte-macrophage colony-stimulating factor, respectively.

### *SF3B1* knockdown

Three non-overlapping small interfering RNAs (siRNAs) targeting *SF3B1* and two different scramble sequences with guanine–cytosine content similar to the siRNA sequences (Stealth Select RNAi, Invitrogen) were used to knock down *SF3B1* in myeloid cell lines. For each transfection, 30 pmol of siRNA and 2 × 10^6^ cells were electroporated in an Amaxa Nucleofector I, using the Amaxa cell optimization kit V (Amaxa, Gaithersburg, MD, USA). Evaluation of green fluorescent protein-positive cells obtained using the pmaxGFP fluorescent expression plasmid confirmed >80% of successfully transfected cells after 24 h. Readout data are reported as mean±s.e.m. Statistical analysis was performed using Student's *t*-test.

### Cell growth assay

Live and dead cells were assessed by trypan blue viability testing and counted using a hemocytometer.

### Cell cycle analysis

Cells were fixed with ice cold methanol, incubated with 40 μg/ml propidium iodide and 10 μg/ml RNaseA, as previously described.^16^ Data were acquired on a BD LSRII flow cytometer (BD Bioscience; Franklin Lakes, NJ, USA) and analyzed using FLOWJO software 7.6.4 (FlowJo, Ashland, OR, USA).

### Erythroid differentiation

To induce erythroid differentiation, K562, TF1 and HEL cells were cultured with hemin 50 μm for 72 h. Erythroid differentiation was studied by analyzing the expression level of γ*-globin* (*HBG1*) using quantitative real-time PCR (qRT-PCR) as described previously.^17^ Expression of the erythroid markers CD36, CD71 and CD235a was evaluated by flow cytometry as described previously.^16^

### Quantitative real-time PCR

Total RNA was reverse transcribed using Retroscript kit (Ambion, Life Technologies, Paisley, UK). The expression levels of *SF3B1*, *ABCB7*, *FTMT*, *HBG1, KLF1* and *B2M* were determined using Assays-on-Demand (Applied Biosystems, Foster City, CA, USA). *B2M* expression levels were used to normalize for differences in input complementary DNA (cDNA). Triplicate samples were run on a LightCycler 480 Real-time PCR system (Roche Diagnostics, Lewes, UK) and expression ratios were calculated using the ddCT method.^18^

### Gene expression profiling

Total RNA (100 ng for each sample) was amplified and labeled with the 3′ IVT Express Kit (Affymetrix, Santa Clara, CA, USA), and hybridized to Affymetrix Human Genome U133 Plus 2.0 GeneChips, as described previously.^19, 20^ CEL files were pre-processed using Robust Multi-chip Average and data analysis was performed using GeneSpring 7.3.1 (Agilent, Santa Clara, CA, USA). Pathway analysis was performed using Ingenuity Pathway Analysis (IPA) 7.5 (Qiagen, Manchester, UK), as previously described.^19^ Analysis of gene set up- or downregulation was performed using Gene Set Enrichment Analysis,^21^ as previously described (false discovery rate<0.1).^2^

### Splicing analysis using human genome exon-junction microarray

Total RNA was DNAse treated (Invitrogen), purified (Agencourt RNA Clean XP) and amplified (100 ng) using Ambion WT Expression Kit (Affymetrix). The cDNA was subsequently fragmented and labeled using WT terminal labeling kit (Affymetrix). Samples were hybridized onto Affymetrix Human genome exon-junction arrays, which on average contain 119 unique probes per gene spanning every exon and known exon–exon junction. CEL files were analyzed using GenoSplice technology (www.genosplice.com).^22, 23^ Data were normalized using quantile normalization. Analysis at the exon level was performed taking into account only exon probes. Analysis at the splicing variant level was performed by taking into account exon–exon junction probes using the FAST DB splicing patterns annotation (release fastdb_2012_2). Unpaired Student's *t*-test was used to determine exon and splicing pattern variation between cells with *SF3B1* knockdown and scramble controls. Genes were considered significantly deregulated when fold change was ⩾1.5 and *P*-value <0.05. Gene Ontology, KEGG and REACTOME analyses of differentially regulated genes were performed using DAVID.^24^

### Splicing analysis using qRT-PCR and Sanger sequencing

Splicing analysis of *TP53* was performed using Sanger sequencing of gel-extracted individual bands from PCR-amplified cDNA. Primers and PCR conditions are listed in Supplementary Table S1.

Splicing analysis of cyclins *CCNA2* and *STK6* was performed using a qRT-PCR on cDNA, using primers specific for splice junctions corresponding to exon inclusion or skipping.^25^ Primers are listed in Supplementary Table S2. *TBP* expression levels were used to normalize for differences in input cDNA.

### RNA sequencing

Bone marrow samples were obtained from MDS patients and healthy controls and CD34^+^ cells isolated using MACS magnetic cell separation columns (Miltenyi Biotec, Germany), as described previously.^19, 20^ RNA extracted from bone marrow CD34^+^ cells obtained from 12 MDS patients and 5 healthy controls was used for deep RNA-Seq. Eight of the 12 MDS cases (four RARS and four RCMD-RS) had *SF3B1* gene mutation, whereas four cases (all RCMD) had no known mutations in splicing factor genes (*SF3B1, SRSF2, U2AF1* or *ZRSR2)* (Supplementary Table S3), as determined by targeted next-generation sequencing data from a previous study.^7^

Total RNA was DNase treated (Invitrogen), purified using XP beads (Beckman Coulter, High Wycombe, UK) and processed (100 ng) using NEBNext Ultra directional mRNA Library prep (NEB, Hitchin, UK) as per the manufacturer's protocol for Illumina with two alterations: custom indexes were designed in house and an additional purification step was performed using XP beads (Beckman Coulter) rather than size selection. Samples were run on HiSeq2000 Illumina sequencing machine (Illumina, San Diego, CA, USA). Sequencing reads were mapped to the human genome using TopHat,^26^ which can handle reads spanning exon–exon boundaries. Data analysis was performed using edgeR^27^ to evaluate the whole transcript expression (false discovery rate<0.05) and with DEXSeq^28^ to evaluate differential exon usage (false discovery rate<0.05). At the expression level, only genes with at least 10 reads in four or more samples were included in the analysis; at the exon level, exons with fewer than 10 reads were excluded. Gender was included as an additional factor in the DEXSeq model testing for differential exon usage. Selected differentially expressed exons were validated using qRT-PCR (Supplemetary Figure S1, Supplementary Information). Integrative Genomics Viewer (IGV) v2.3 (http://www.broadinstitute.org/igv/) was used for visualization of the sequence reads. Pathway analysis was performed using IPA and Gene Set Enrichment Analysis, as described above. The data discussed in this publication have been deposited in NCBI's Gene Expression Omnibus and are accessible through GEO Series accession number GSE63569.

## Results

### *SF3B1* knockdown inhibits cell growth, induces cell cycle arrest and impairs erythroid differentiation

**Figure 1:**
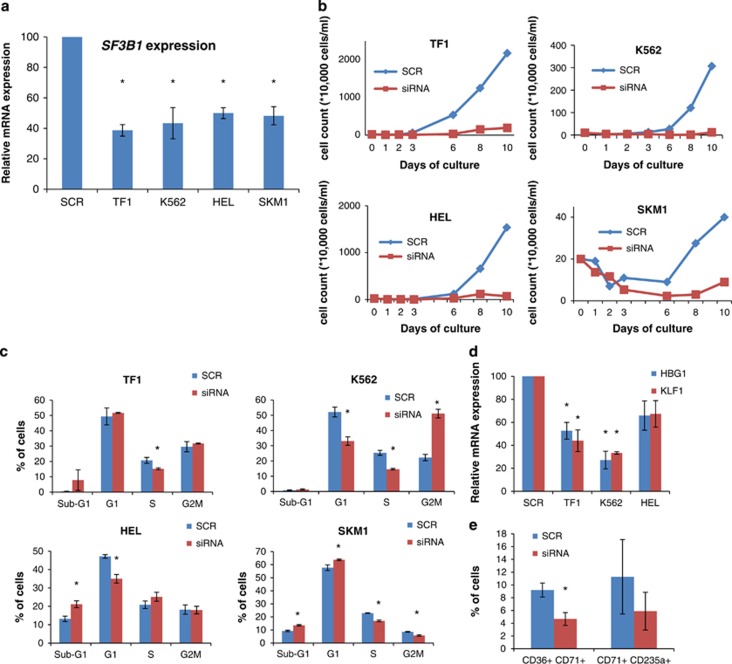
Effects of *SF3B1* knockdown in myeloid cell lines. Each cell line transfected with siRNA targeting *SF3B1* was compared with the corresponding cell line transfected with the scramble control. (**a**) *SF3B1* mRNA expression measured 3 days after knockdown. (**b**) Growth curves of cells with *SF3B1* knockdown, compared with cells transfected with the scramble control, as assessed by trypan blue exclusion. (**c**) Cell cycle analysis of cell lines following *SF3B1* knockdown. (**d**) Erythroid differentiation in myeloid cell lines with *SF3B1* knockdown treated with 50 μm hemin, as measured by *HBG1* and *KLF1* expressions relative to the scramble control. (**e**) Percentage of CD36+CD71+ and CD71+CD235a+ populations in K562 cells with *SF3B1* knockdown compared with the scramble control. Results in subpanels **a**–**d** were obtained from scramble *n*=2 and *SF3B1* siRNA as follows: **a**, *n*=3; **b**, *n*=3; **c**, *n*=3 for SKM1 and TF1, *n*=6 for HEL and *n*=9 for K562; **d**, *n*=3 for TF1 and HEL, *n*=6 for K562. Results in subpanel **e** were obtained from scramble *n*=4 and *SF3B1* siRNA *n*=4. *P<0.05.

*SF3B1* was knocked down using siRNA technology in four myeloid cell lines (TF1, K562, HEL and SKM1) that we found to be wild type for *SF3B1* (Supplementary Information), resulting in a significant decrease in expression ranging between 50 and 60% (Figure 1a).

Cell growth was inhibited in all four cell lines with *SF3B1* knockdown in comparison with the scramble control (Figure 1b). Cell cycle arrest in different phases was detected in different myeloid cell lines with *SF3B1* knockdown (Figure 1c). K562 cells showed a significant G2M cell cycle arrest with a concomitant reduction in the percentage of cells in the G1 and S phase. TF1 cells showed a significant decrease in the percentage of cells in the S phase. HEL cells showed a significant decrease in the percentage of cells in the G1 phase and a significant increase in the sub-G1 cell population, indicating increased apoptosis. Similarly, SKM1 cells showed a significant decrease in the percentage of cells in S and G2M phase with a concomitant increase in the percentage of cells in the sub-G1 and G1 phase, indicating cell cycle arrest in the G1 phase and an increase in apoptosis (Figure 1c).

Three cell lines (TF1, K562 and HEL) with *SF3B1* knockdown were cultured with hemin to induce erythroid differentiation. We assessed the expression of the erythroid differentiation markers *HBG1* and *KLF1* using qRT-PCR. A significant reduction in the expression of *HBG1* and *KLF1* was observed in TF1 and K562 cell lines with *SF3B1* knockdown (Figure 1d). In addition, we observed a reduction in the percentage of CD36+CD71+ and CD71+CD235a+ erythroid populations (significant for the CD36+CD71+ population) in K562 cells with *SF3B1* knockdown compared with the scramble control (Figure 1e), suggesting that normal *SF3B1* function is required for erythroid differentiation.^17^

**Figure 2:**
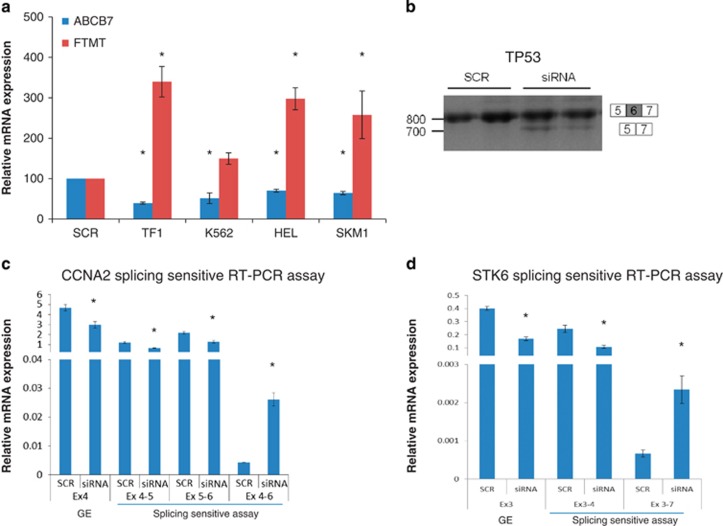
Effects of *SF3B1* knockdown on gene expression and splicing. (**a**) *ABCB7* and *FTMT* expression levels in TF1, K562, HEL and SKM1 cells with *SF3B1* knockdown, as measured by qRT-PCR 48 h post transfection. Each cell line transfected with siRNA targeting *SF3B1* was compared with the corresponding cell line transfected with the scramble control. (**b**) Reverse transcription-PCR of *TP53* exons 5–7 showing aberrant splicing in K562 cells with *SF3B1* knockdown (siRNA) compared with scramble (SCR). (**c**, **d**). qRT-PCR analysis using primers that monitor general gene expression (GE) in a constitutive exon (Ex4 of *CCNA2* and Ex3 of *STK6*) or primers specific for splice junctions corresponding to exon inclusion or skipping in the cyclin A2 (*CCNA2*) and Aurora Kinase A (*STK6*) genes in K562 cells. Cells with *SF3B1* knockdown (siRNA) show alternative splicing events. Results in subpanel **a** were obtained from scramble *n*=3 and *SF3B1* siRNA *n*=4. Results in subpanel **c**–**d** were obtained from scramble *n*=2 and *SF3B1* siRNA *n*=3. *P<0.05.

RARS is characterized by *FTMT* accumulation and low expression levels of the iron transporter *ABCB7*.^17, 29^ We have previously shown that *SF3B1* knockdown leads to decreased *ABCB7* expression and increased *FTMT* expression in K562 cells.^17^ In this study, we have extended these observations to the other three myeloid cell lines investigated (Figure 2a). In addition, restoration of *SF3B1* expression to normal levels after 10 days of culture was followed by restoration of *ABCB7* expression levels to normal (Supplementary Figure S2).

Taken together, these data show that *SF3B1* knockdown results in inhibition of cell growth, induction of cell cycle arrest and impairment of erythroid differentiation in myeloid cell lines.

### *SF3B1* knockdown alters gene expression

To evaluate the effects of *SF3B1* knockdown on global gene expression, gene expression profiling was performed in the four myeloid cell lines. For each cell line, we compared the expression profiles of cells treated with two different siRNAs targeting *SF3B1* with those of cells treated with a scramble control, 48 h post transfection.

We identified many genes that were up- or downregulated by >2-fold in each cell line treated with *SF3B1* siRNAs (Supplementary Figure S3A and S3B). Four genes were upregulated (*TFDP1, LOC100505759, MKRN1* and *WRNIP1*) and five genes were downregulated (*ZC3H7A, CREBZF, SGK494, WSB1* and two probesets for *SF3B1*) in all four cell lines with *SF3B1* knockdown.

Pathway analysis was performed on the up- and downregulated genes in each cell line with *SF3B1* knockdown using IPA. Significant deregulation of pathways related to cell cycle regulation was observed in all cell lines and of mTOR signaling and AMPK signaling pathways in three cell lines (Table 1). We performed Gene Set Enrichment Analysis to identify pathways and processes showing coordinated up- or downregulation. Upregulated gene sets include p53 signaling in K562 and SKM1 cells, and several gene sets associated with regulation of transcription, spliceosome and splicing in K562 cells (Supplementary Table S4). Downregulated gene sets associated with the mitochondrial function were found in K562 and TF1 cells, and with cell cycle regulation in SKM1 and HEL cells (Supplementary Table S4). These data show that *SF3B1* knockdown in the cell lines studied results in deregulation of many genes and pathways including cell cycle and RNA processing.

### *SF3B1* knockdown impact on splicing

The genome-wide effects of *SF3B1* knockdown on splicing were investigated in two myeloid cell lines (K562 and TF1) using human genome exon-junction arrays. The splicing profile of cells with *SF3B1* knockdown (using two different siRNA per cell line) was compared with that of cells transfected with the scramble control.

We observed 2027 differentially expressed exons from 1419 genes, and 507 significant differentially regulated splicing variants (including exon skipping, intron retention and alternative splice sites) of 384 genes in cells with *SF3B1* knockdown. For example, we observed differential exon usage of *CDC7* and *SRSF11* in the data from both the cell lines, and of *TP53* in TF1 cells. We found a significant overrepresentation of 3′ acceptor splice sites affected by alternative splicing events compared with 5′ donor splice sites (5:1 ratio, *P*=0.0027, *χ*^2^ test with Yates' correction), consistent with the known role of SF3B1 in the recognition of 3′ splice sites. Gene ontology analysis was performed using DAVID (http://david.abcc.ncifcrf.gov/), and many themes showed significant enrichment of genes affected at the level of exon usage and splice variants (Table 2). Cell cycle and RNA degradation were found to be consistently deregulated at both levels (Table 2). We investigated *TP53* differential exon usage by PCR amplification and Sanger sequencing of gel-extracted bands. We observed exon skipping that was present in the *SF3B1* knockdown cells only (Figure 2b).

We also investigated whether two cell cycle genes (*CCNA2* and *STK6*) previously shown to be aberrantly spliced in HeLa cells with *SF3B1* knockdown,^25^ were also aberrantly spliced in K562 cells with *SF3B1* knockdown in our study. Consistent with the finding in HeLa cells, we observed aberrant splicing of these genes using a qRT-PCR strategy as described previously (Figure 2c and d).^25^

### RNA-Seq in HSPC from MDS patients with *SF3B1* mutations

To gain insight into the spectrum of genes that are deregulated or aberrantly spliced in association with *SF3B1* mutation in the hematopoietic stem and progenitor cells (HSPC) of MDS patients, we used deep RNA-Seq to compare the transcriptome of bone marrow CD34^+^ cells from eight MDS patients with *SF3B1* mutation (*SF3B1* mutants), four MDS patients with no known splicing mutation (wild type) and five healthy controls (control) (Supplementary Table S3). Using IGV, we evaluated the expression of the *SF3B1* alleles in *SF3B1* mutant cases and observed a range of 45–52% of mutant allele frequency, indicating that both wild-type and mutant alleles were equally expressed (Supplementary Table S3, Supplementary Figure S4).

We used edgeR to perform differential gene expression analysis of *SF3B1* mutants versus wild type and control. At the whole transcript level, we observed a total of 526 genes (253 upregulated and 273 downregulated) significantly differentially expressed in *SF3B1* mutants in comparison with wild type (Supplementary Table S5). In the comparison of *SF3B1* mutants with control, we found 1823 significantly differentially expressed genes (646 upregulated and 1177 downregulated) (Supplementary Table S6). Genes linked to the pathogenesis of RARS and RCMD-RS, such as *ALAS2* and *ABCB7*, were deregulated (*ALAS2* upregulated and *ABCB7* downregulated) in both comparisons of *SF3B1* mutant with wild type and control. We also observed upregulation of the mitochondrial genes *SLC25A37* when comparing *SF3B1* mutant with control and *GLRX5* in both comparisons of *SF3B1* mutant with wild type and control.

We performed an analysis using 121 genes either known to be expressed in erythroid cells^30^ or described as erythroid transcription factors in the literature. We found 42 differentially expressed genes (37 upregulated and 5 downregulated) when comparing *SF3B1* mutant with control. These included heme biosynthetic enzymes (for example, *ALAS2*, *ALAD*, *FECH* and *UROD*), globin genes (for example, *HBQ1*, *HBA2*, *HBB* and *HBA1*) and transcription factors (for example, *GATA1*, *GATA2* and *KLF1*). In the comparison of *SF3B1* mutant with wild type, we found a total of 32 differentially expressed genes (31 upregulated and 1 downregulated) of which 28 were overlapping with the differentially expressed genes found when comparing *SF3B1* mutant with control.

We then performed pathway analysis on the significantly differentially expressed genes using IPA. Many pathways, including heme biosynthesis, mitotic roles of polo-like kinase and TNFR2 signaling, were significantly deregulated in the comparison of *SF3B1* mutant with wild type (Table 3). When comparing *SF3B1* mutant with control, significantly deregulated pathways included apoptosis signaling, p53 signaling, cell cycle regulation and heme degradation (Supplementary Table S7). We next performed Gene Set Enrichment Analysisand many gene sets showing significant enrichment were identified in *SF3B1* mutant versus wild type and control. Upregulated gene sets included several that were related to mitochondrial function, cell cycle checkpoints and mRNA splicing. In the comparison of *SF3B1* mutant with control cases, downregulated gene sets included several that were related to cell differentiation and apoptosis (Supplementary Table S8). Many of these deregulated pathways and gene sets are relevant to the known pathophysiology of MDS and in particular of RARS and RCMD-RS.

**Figure 3:**
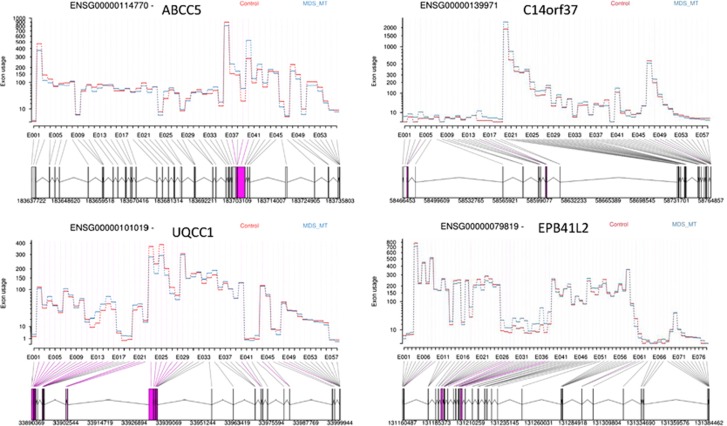
Examples of genes showing significant differential exon usage between MDS patients with *SF3B1* mutation in comparison with control, obtained from RNA-Seq data analysis using DEXSeq. The graphs show some of the top ranking genes with significant differential exon usage. The exons highlighted in purple represent the significant differential exon usage.

**Figure 4:**
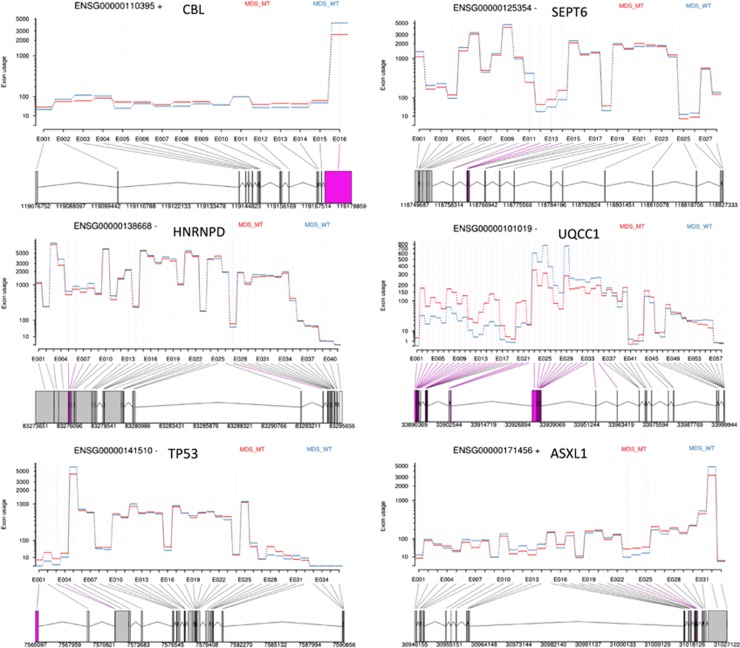
Examples of genes showing significant differential exon usage in MDS patients with *SF3B1* mutation in comparison with wild type, obtained from RNA-Seq data analysis using DEXSeq. The graphs show some of the top ranking genes with significant differential exon usage. The exons highlighted in purple represent the significant differential exon usage.

DEXSeq was used to perform differential exon usage analysis of the RNAseq data to evaluate aberrantly spliced genes. At the exon level, we observed a total of 3506 exons (corresponding to 1924 genes) significantly differentially expressed in *SF3B1* mutant compared with control (Table 4, Figure 3, Supplementary Table S9). Differential exon usage was observed in at least one exon of genes known to be involved in MDS pathophysiology (*TP53* and *EZH1*), erythroid genes (*ALAD, UROD* and *EPB42*) and genes associated with cell cycle (*AURKB* and *CRNDE*) and RNA processing (*RBM5, RBM25, PRPF40A* and *HNRNPD*). When comparing *SF3B1* mutant with wild type cases, we found 3097 significantly differentially expressed exons (corresponding to 2022 genes) (Table 5, Figure 4, Supplementary Table S10). We found differential exon usage in at least one exon of genes involved in MDS pathophysiology (*CBL, ASXL1* and *DNMT3A*), mitochondrial function (*ALAS2, NDUFAF6*), erythroid differentiation (*NFE2L2, PPOX* and *HMBS*) and mRNA processing (*HNRNPD, U2AF2* and *PRPF8*). Interestingly, *UQCC1*, a gene involved in mitochondrial biogenesis^31^ and showing abnormal splicing in *SF3B1* mutant cases in uveal melanoma,^32^ showed differential exon usage and upregulation in *SF3B1* mutant cases compared with wild type and control in our study.

To identify pathways affected by differential exon usage, we performed pathway analysis on the genes showing significantly differentially expressed exons using IPA. In the comparison of *SF3B1* mutant with wild type and control, we observed many pathways to be affected, including cell cycle, heme biosynthesis, DNA damage response, mitochondrial and haematopoietic progenitor cells pathway (Supplementary Table S11 and S12). Using DAVID functional annotation tool, we observed significant enrichment of biological themes including alternative splicing, RNA binding, mitochondrion, spliceosome and cell cycle.

Recently, a role for SF3B1 in the maintenance of genomic stability has also been reported where it functions in a DNA damage-induced mRNA splicing complex with BRCA1 and BCLAF1.^33^ Given that deregulation of the DNA damage response pathway was highlighted by the IPA pathway analysis, we performed an analysis using genes regulated by the BRCA1–BCLAF1–SF3B1 complex. Several genes regulated by this complex showed differential exon usage in *SF3B1* mutant compared with control (including *NUMA1, RB1, CHUK* and *ABL1*) and compared with wild type (*NUMA1*, *PIAS1*, *SMAD4*, *BIRC2* and *PTK2)* (Supplementary Table S13). The overrepresentation of genes regulated by the BRCA1–BCLAF1–SF3B1 complex was significant in *SF3B1* mutant compared with control (*P*<0.001) and compared with wild type (*P*=0.0498, hypergeometric test). We also found many genes to be affected at the transcript level, including *BIRC3*, *BCL2A1, GYPB, HBB* and *HBBP1* when comparing *SF3B1* mutant with wild type and control (Supplementary Table S13).

## Discussion

The identification of frequent somatic mutations of *SF3B1* in MDS patients with ring sideroblasts suggests a direct correlation between the presence of mutations and this particular phenotype.^2, 11, 13^ However, the mechanism by which *SF3B1* mutation leads to MDS with ring sideroblasts remains to be elucidated.

To illuminate the role of *SF3B1* mutation in MDS pathophysiology, we have determined the effects of *SF3B1* disruption on cell growth and gene expression in human hematological cells. First, we investigated the effects of *SF3B1* knockdown on cell function and gene expression in myeloid cell lines. Second, we used RNA-Seq to study the global gene expression changes and splicing abnormalities associated with the presence of *SF3B1* mutations in the HSPC of MDS patients.

We have shown that *SF3B1* knockdown in four myeloid cell lines resulted in inhibition of cell growth and disruption of the cell cycle. It has recently been reported that Sf3b1^+/−^ mice showed reduced numbers of HSC and compromised reconstitution capacity in lethally irradiated mice.^14, 15^ Thus, reduced expression of *SF3B1* appears to result in impaired cell growth in hematopoietic cells. Similar observations have been made following reduced expression of other splicing factor genes, including *U2AF1*.^3, 4, 34^

We used two different microarray platforms to identify genes deregulated at the transcriptional and exon level by *SF3B1* knockdown in myeloid cell lines. Gene expression profiling identified many deregulated genes, with four genes consistently upregulated, including *TFDP1,* and five genes downregulated, including *CREBZF,* in all four cell lines with *SF3B1* knockdown, several of which have a role in the control of cell growth. *TFDP1* is involved in the control of transcriptional activity of G1/S cell cycle checkpoint genes,^35^ and *CREBZF* is a potent suppressor of cell growth, the effects of which are mediated through the tumor suppressor p53.^36^ Next, we employed exon-junction arrays to study the transcriptome in K562 and TF1 cells. We identified many differentially expressed exons and differentially regulated splicing variants in cells with *SF3B1* knockdown. For example, we observed differential exon usage of the *CDC7* and *SRSF11* genes, involved in cell cycle regulation and splicing, respectively, and of the *TP53* gene. Several pathways including cell cycle, RNA processing, mitochondrion and apoptosis/p53 pathway were consistently deregulated in the cell lines with *SF3B1* knockdown.

In summary, our data on myeloid cell lines suggest that the phenotype observed in cells with *SF3B1* knockdown is mediated by aberrant splicing and expression of target genes involved in key biological processes.

RNA-Seq is the method of choice for a comprehensive analysis of global gene expression and splicing. In all relevant studies published to date, RNA-Seq has been performed on unfractionated bone marrow mononuclear cells from a small number of MDS patients with *SF3B1* mutations (*n*⩽3 per study), revealing interesting data.^11, 37, 38^ MDS arise in the HSC, and it is thus of critical importance that the effects of *SF3B1* mutation on the transcriptome are studied in the cell of origin. In this study, we performed RNA-Seq on purified bone marrow CD34^+^ cells from 12 MDS patients, eight with ring sideroblasts and *SF3B1* mutation and four without mutation in other splicing factor genes, and from five healthy controls.

We identified many significantly differentially expressed genes at the transcript level and the exon level when comparing *SF3B1* mutant with wild type and control. CD34^+^ cells from RCMD-RS and RARS patients display a particular expression profile of mitochondria-related genes;^17, 20^ we observed upregulation of *ALAS2* (heme biosynthesis enzyme) and downregulation of *ABCB7* (involved in the transport of iron from the mitochondria to the cytoplasm) in *SF3B1* mutants compared with wild type and control, in agreement with our previous reports.^17, 20^ *SLC25A37*, encoding a mitochondrial iron importer, and *GLRX5*, encoding another mitochondrial protein, were both significantly upregulated in CD34^+^ cells of *SF3B1* mutants compared with wild type and control. A recent study showed *SLC25A37* mRNA upregulation in *SF3B1* mutant bone marrow mononuclear cells of three MDS patients with RARS/-T.^38^ Evidence is thus mounting to suggest that upregulation of the iron importer *SLC25A37* and downregulation of iron exporter *ABCB7* may be linked to the increased mitochondrial iron accumulation observed in MDS patients with ring sideroblasts.^39^ Deregulation of these genes could also be consistent with an impaired ability of mitochondrial pathways to use iron that may result in an attempt to increase iron availability.

Importantly, we observed many genes associated with porphyrin and heme biosynthesis showing differential exon usage in *SF3B1* mutant cases, indicating altered splicing. These include *ALAD* and *UROD* when comparing *SF3B1* mutant with control and *ALAS2* and *PPOX* when comparing with wild type. We suggest that the aberrant expression and exon usage of mitochondrial and heme-related genes in the CD34^+^ cells of MDS patients with *SF3B1* mutation has a role in ring sideroblast formation and abnormal iron homeostasis observed in this patient group. Intriguingly, the most common congenital sideroblastic anemia (X-linked sideroblastic anemia) results from an *ALAS2* mutation.^40^

The relative expression levels of the transcription factors *GATA1* and *GATA2* differed from normal in the CD34^+^ cells of MDS patients with *SF3B1* mutation. The dynamic and strictly regulated change of expression from *GATA2* to *GATA1* during erythropoiesis has a crucial role,^41^ and any alteration of this pattern may be predicted to lead to aberrant erythropoiesis. We suggest that the aberrant expression and splicing of erythroid-related genes observed in the CD34^+^ cells of MDS patients with *SF3B1* mutation may have a role in the ineffective erythropoiesis found in these patients.

In a previous study, RNA-Seq was used to compare the transcriptome of bone marrow mononuclear cells of two MDS patients with *SF3B1* mutation to that of one healthy control.^11^ One hundred and thirty genes showed significant differential expression, 28 of which overlap with the genes differentially expressed between *SF3B1* mutant and control in our study of CD34^+^ cells. These genes include *MAP3K8* and *CLEC5A*, which have been previously shown to be downregulated in MDS.^19, 42^ Furthermore, 350 genes showed differential exon usage, and 52 of these were in common with the genes showing differential exon usage in our study of CD34^+^ cells. The overlapping genes include *EZH1*, *ASXL1, CBL1* and *SMAD4*.

The mutant forms of *ASXL1*, *CBL* and *TP53* are known to have an important role in the molecular pathogenesis of MDS, and intriguingly all these genes were found to be alternatively spliced in the HSPC of MDS patients harboring *SF3B1* mutations.

A recent study identified a DNA damage-induced BRCA1 protein complex containing BCLAF1 and SF3B1 (BRCA1–BCLAF1–SF3B1 complex).^33^ In response to DNA damage, the complex regulates pre-mRNA splicing of genes involved in DNA damage signaling and repair and hence affects their transcription and pre-mRNA maturation.^33^ Importantly, we found many of the genes regulated by this complex to be differentially expressed and to show differential exon usage in our study. Genes showing differential transcript level changes included *BIRC3* and *BCL2A1*, whereas *NUMA1* showed differential exon usage when comparing *SF3B1* mutant with wild type and control. When comparing *SF3B1* mutant with wild type, we also found differential exon usage of the *PIAS1* gene, a DNA damage response regulator.^43, 44^ It has been demonstrated that abrogation of members of this complex including BRCA1 and BCLAF1 results in genomic instability,^33^ a common feature of malignant cells. In MDS patients with mutant *SF3B1*, the function of this complex may be impaired, with possible downstream effects on the efficiency of DNA damage repair.

Many genes involved in RNA splicing and processing were found to show differential transcript levels, as well as differential exon usage in our study, including *HNRNPD, U2AF2, PRPF8* and *RBM25*. A recent study showed RNA processing genes to be misspliced in the bone marrow mononuclear cells of MDS/AML patients with *U2AF1* mutation.^45^ Intriguingly, there is limited overlap between the genes misspliced as a result of *U2AF1* mutation and the RNA processing/splicing genes deregulated/aberrantly spliced in our *SF3B1* mutant cases in both comparisons of *SF3B1* mutant with wild type and control, suggesting that this process may be affected by different splicing mutations even if their target genes are distinct.

A recent study showed that defects in the splicing factor gene *PRPF8* result in missplicing in myeloid malignancies and are associated with the presence of ring sideroblasts in advanced MDS and AML.^46^ Interestingly, we observed differential exon usage of *PRPF8* in *SF3B1* mutant cases compared with control in our study, indicating a link between *SF3B1* and *PRPF8* and the ring sideroblast phenotype. Our finding of differential exon usage of multiple RNA processing/splicing genes in the HSPC of cases with *SF3B1* mutation points towards an exacerbation of aberrant splicing, with a wider number of downstream target genes affected. Emerging evidence from our study on *SF3B1* and the study on *U2AF1*^45^ thus supports the hypothesis that in MDS patients harboring splicing factor mutations, there is widespread disruption of the splicing machinery as a consequence of downstream effects of the mutant protein. Loss-of-function mutations in zebrafish *Prpf8* have been shown to result in missplicing of *TP53*.^47^ We found *TP53* to be aberrantly spliced in MDS patients with *SF3B1* mutation compared with wild type and control.

Splicing factor mutations, including *SF3B1*, are considered to be founder mutations and must confer a selective growth advantage to the HSC, but how this occurs remains a mystery. We have identified differential splicing in several genes known to have a role in MDS pathogenesis, including *TP53* and *ASXL1*, as well as genes involved in DNA damage repair, in the HSPC of MDS patients harboring *SF3B1* mutations. We suggest that the aberrant splicing of such genes may confer oncogenic properties that help drive the malignant process. In this study, we have determined the target genes of *SF3B1* mutations in the HSPC of MDS patients with ring sideroblasts, shedding light on the mechanisms underlying this phenotype.

## Figures and Tables

**Table 1:** List of significant deregulated pathways in cell lines obtained using IPA

*TF1*	*SKM1*	*K562*	*HEL*
Selenoamino acid metabolism	Estrogen-mediated S-phase entry	AMPK signaling	Estrogen-mediated S-phase entry
Mitochondrial dysfunction	Pentose phosphate pathway	HMGB1 signaling	Cyclins and cell cycle regulation
AMPK signaling	Cell cycle: G1/S checkpoint regulation	mTOR signaling	PI3K signaling in B lymphocytes
Regulation of eIF4 and p70S6K signaling	Small-cell lung cancer signaling	Polyamine regulation in colon cancer	mTOR signaling
mTOR signaling	Lysine biosynthesis	Cell cycle regulation by BTG family proteins	Breast cancer regulation by stathmin1
Role of NANOG in mammalian embryonic stem cell pluripotency	Actin cytoskeleton signaling	ILK signaling	Purine metabolism
Cyclins and cell cycle regulation	Chronic myeloid leukemia signaling	Cyclins and cell cycle regulation	Glioma signaling
Polyamine regulation in colon cancer	Glioma signaling	Ceramide signaling	Aldosterone signaling in epithelial cells
Assembly of RNA polymerase II complex	Assembly of RNA polymerase III complex	ErbB signaling	fMLP signaling in neutrophils
	Signaling by Rho family GTPases	TNFR1 signaling	Cell cycle: G1/S checkpoint regulation
		Glucocorticoid receptor signaling	Non-small cell lung cancer signaling
		Glycine, serine and threonine metabolism	Growth hormone signaling
		Role of CHK proteins in cell cycle checkpoint control	Small-cell lung cancer signaling
		VDR/RXR activation	Protein ubiquitination pathway
		Lysine degradation	Nitric oxide signaling in the cardiovascular system
		Tight junction signaling	AMPK signaling
		Ubiquinone biosynthesis	Glioblastoma multiforme signaling
		Mitotic roles of polo-like kinase	
		Wnt/β-catenin signaling	
		PI3K/AKT signaling	
		Renal cell carcinoma signaling	
		Regulation of eIF4 and p70S6K signaling	
		TNFR2 signaling	
		Fructose and mannose metabolism	
		Telomerase signaling	

Gene expression profiling was performed in all four cell lines with *SF3B1* knockdown, and genes showing >2-fold change were used for the analysis.

**Table 2:** Pathway analysis using human genome exon-junction array data at two different levels: differential exon usage and different splicing variant

*Pathways affected by diferential exon usage*		*Pathways affected by differential splice variant usage*	
*Pathway description*	P*-value*	*Pathway description*	P*-value*
*KEGG pathway*			
Ubiquitin-mediated proteolysis	1.82E−05	RNA degradation	7.85E−05
Cell cycle	1.02E−04	Oocyte meiosis	4.23E−03
Spliceosome	3.22E−04	Cell cycle	8.45E−03
Oocyte meiosis	3.88E−04	Progesterone-mediated oocyte maturation	2.17E−02
Phosphatidylinositol signaling system	8.92E−04	Aminoacyl-tRNA biosynthesis	4.04E−02
Inositol phosphate metabolism	1.68E−03		
RNA degradation	2.30E−02		
Ribosome	2.58E−02		
One carbon pool by folate	2.65E−02		
Aminoacyl-tRNA biosynthesis	2.91E−02		
Selenoamino acid metabolism	3.93E−02		
Insulin signaling pathway	4.03E−02		
			
*REACTOME pathway*			
Cell cycle, mitotic	1.19E−09	Cell cycle, mitotic	4.43E−05
Gene expression	1.47E−04	Gene expression	1.29E−02
DNA repair	1.75E−03	Cell cycle checkpoints	2.20E−02
APC-Cdc20-mediated degradation of Nek2A	9.97E−03		
Cell cycle checkpoints	1.13E−02		
Transcription	1.22E−02		
Signaling by Wnt	2.91E−02		
Signaling by NGF	3.63E−02		
Cdc20:Phospho-APC/C-mediated degradation of cyclin A	4.70E−02		

**Table 3:** Pathway analysis (IPA) of the significant differentially expressed genes between *SF3B1* mutant and wild type obtained using edgeR

*Ingenuity canonical pathways*	P*-value*	*Ratio*	*Molecules*
Lymphotoxin β receptor signaling	0.00074131	9.68E−02	NFKBID, BCL2L1, CASP3, RELB, CXCL1 and TRAF1
Agranulocyte adhesion and diapedesis	0.001071519	5.73E−02	CXCL3, PODXL2, CCL4,CLDN19, CXCL14, CXCL1, CCL3L1/CCL3L3, MMP2, CXCL2, MYH7B and CCL4L1/CCL4L2
Heme biosynthesis II	0.001096478	1.67E−01	FECH, ALAS2, CPOX and HMBS
Hepatic fibrosis/hepatic stellate cell activation	0.00162181	5.81E−02	CXCL3, LEPR, IL6R, MMP2, LBP, IL6, MYH7B, AGTR1 and PGF
Communication between innate and adaptive immune cells	0.001995262	6.25E−02	CCL4, TLR7, CCL3L1/CCL3L3, CD83, IGHG1, IGHA1 and IL6
Colorectal cancer metastasis signaling	0.003467369	4.48E−02	BCL2L1, ADCY9, CDH1, JUN, CASP3, PTGER3, DIRAS3, IL6R, TLR7, MMP2, IL6 and PGF
IL-17A signaling in fibroblasts	0.004570882	1E−01	NFKBID, JUN, IL6 and NFKBIZ
Toll-like receptor signaling	0.004570882	7.81E−02	JUN, TLR7, TNFAIP3, LBP and TRAF1
Differential regulation of cytokine production in macrophages and T-helper cells by IL-17A and IL-17F	0.004786301	1.67E−01	CCL4, CXCL1 and IL6
Granulocyte adhesion and diapedesis	0.007762471	4.95E−02	CXCL3, CCL4, CLDN19, CXCL14, CXCL1, CCL3L1/CCL3L3, MMP2, CXCL2 and CCL4L1/CCL4L2
Airway pathology in chronic obstructive pulmonary disease	0.009772372	1.82E−01	CXCL3 and MMP2
TNFR2 signaling	0.016595869	8.82E−02	JUN, TNFAIP3 and TRAF1
Heme bosynthesis from uroporphyrinogen-III I	0.018620871	1.82E−01	FECH and CPOX
Aryl hydrocarbon receptor signaling	0.024547089	4.09E−02	TGM2, CCNE1, GSTM2, ALDH1A1, JUN, NQO2 and IL6
NRF2-mediated oxidative stress response	0.025118864	4.1E−02	GSR, JUN, GSTM2, NQO2, DNAJC6, JUND, FOSL1 and ABCC4
Role of IL-17A in psoriasis	0.025703958	1.43E−01	CXCL3 and CXCL1
Gα12/13 signaling	0.028840315	4.72E−02	BTK, CDH7, CDH1, JUN, MEF2D and CDH11
Tetrapyrrole biosynthesis II	0.029512092	1.43E−01	ALAS2 and HMBS
CDK5 signaling	0.033884416	5.15E−02	FOSB, PPP1CC, ADCY9, PPM1J and EGR1
CD40 signaling	0.033884416	5.63E−02	JUN, TNFAIP3, MAPKAPK2 and TRAF1
Mitotic roles of polo-like kinase	0.041686938	5.41E−02	PLK3, PPM1J, ANAPC13 and CCNB1
Adenine and adenosine salvage III	0.042657952	1.18E−01	PNP and ADAT3
Tryptophan degradation to 2-amino-3-carboxymuconate semialdehyde	0.046773514	1.11E−01	HAAO and KYNU
T-helper cell differentiation	0.047863009	5.56E−02	STAT4, IL6R, IL6 and RORC

**Table 4:** Top ranking genes showing differential exon usage between *SF3B1* mutant and control, obtained from RNA sequencing data analysis using DEXSeq

*Gene ID*	*Exon ID*	*Gene name*	*Chromosome*	*Strand*	P*-value*	P_*adj*_	*FC*
ENSG00000125354	E011	SEPT6	X	−1	1.67E−78	5.19E−73	0.822522658
ENSG00000111843	E006	TMEM14C	6	1	2.80E−51	4.36E−46	0.803897136
ENSG00000131669	E006	NINJ1	9	−1	1.09E−33	1.13E−28	0.835812796
ENSG00000088986	E014	DYNLL1	12	1	1.21E−28	9.43E−24	0.913566493
ENSG00000223865	E018	HLA-DPB1	6	1	3.05E−27	1.90E−22	1.357267219
ENSG00000101019	E025	UQCC1	20	−1	1.71E−26	8.88E−22	0.889537433
ENSG00000132199	E025	ENOSF1	18	−1	2.57E−24	1.14E−19	0.905515696
ENSG00000125991	E049	ERGIC3	20	1	4.11E−23	1.42E−18	0.870728412
ENSG00000101019	E024	UQCC1	20	−1	2.01E−22	6.25E−18	0.878172451
ENSG00000118495	E010	PLAGL1	6	−1	2.78E−22	7.85E−18	1.753766932
ENSG00000266086	E005	RP11-159D12.5	17	−1	1.43E−18	3.43E−14	1.051487592
ENSG00000119777	E023	TMEM214	2	1	1.35E−18	3.43E−14	0.724952987
ENSG00000130066	E011	SAT1	X	1	2.63E−17	5.83E−13	1.224469811
ENSG00000101019	E029	UQCC1	20	−1	3.49E−17	7.24E−13	0.987866168
ENSG00000141425	E015	RPRD1A	18	−1	2.14E−16	3.91E−12	1.151614234
ENSG00000268400	E058	CTD-3214H19.4	19	1	2.08E−16	3.91E−12	0.720165623
ENSG00000127586	E055	CHTF18	16	1	2.45E−16	4.24E−12	0.83461565
ENSG00000130066	E015	SAT1	X	1	4.80E−16	7.86E−12	1.112053709
ENSG00000160789	E072	LMNA	1	1	1.00E−15	1.56E−11	0.74131183
ENSG00000130066	E010	SAT1	X	1	1.22E−15	1.81E−11	1.175909464
ENSG00000119777	E024	TMEM214	2	1	6.13E−15	8.66E−11	0.769414939
ENSG00000266086	E004	RP11-159D12.5	17	−1	1.51E−14	2.04E−10	1.073999383
ENSG00000168675	E009	LDLRAD4	18	1	4.10E−14	5.10E−10	0.882283199
ENSG00000160789	E071	LMNA	1	1	6.34E−14	7.58E−10	0.907377148
ENSG00000214021	E019	TTLL3	3	1	1.13E−13	1.30E−09	1.027729333
ENSG00000196576	E041	PLXNB2	22	−1	1.59E−13	1.77E−09	0.53703246
ENSG00000166508	E021	MCM7	7	−1	2.21E−13	2.37E−09	1.034956343
ENSG00000114770	E040	ABCC5	3	−1	2.75E−13	2.79E−09	1.290748901
ENSG00000196365	E028	LONP1	19	−1	2.78E−13	2.79E−09	0.876937237
ENSG00000130066	E002	SAT1	X	1	3.59E−13	3.49E−09	0.967812734
ENSG00000102119	E017	EMD	X	1	3.76E−13	3.55E−09	1.050545479
ENSG00000130066	E014	SAT1	X	1	5.55E−13	5.08E−09	1.168469304
ENSG00000266086	E006	RP11-159D12.5	17	−1	5.72E−13	5.08E−09	1.073091463
ENSG00000055609	E005	KMT2C	7	−1	6.09E−13	5.26E−09	1.089688525
ENSG00000228315	E065	GUSBP11	22	−1	6.70E−13	5.63E−09	1.125912723
ENSG00000156860	E023	FBRS	16	1	7.27E−13	5.95E−09	1.10075677
ENSG00000268400	E071	CTD-3214H19.4	19	1	8.37E−13	6.67E−09	1.139015436
ENSG00000214021	E065	TTLL3	3	1	1.15E−12	8.34E−09	1.269221488
ENSG00000122566	E004	HNRNPA2B1	7	−1	1.10E−12	8.34E−09	1.160615984
ENSG00000101557	E035	USP14	18	1	1.13E−12	8.34E−09	1.043528763

Genes are ranked by adjusted *P*-value (*P*_adj_).

**Table 5:** Top ranking genes showing differential exon usage between *SF3B1* mutant and wild type, obtained from RNA sequencing data analysis using DEXSeq

*Gene ID*	*Exon ID*	*Gene name*	*Chromosome*	*Strand*	P*-value*	P_*adj*_	*FC*
ENSG00000125354	E011	SEPT6	X	−1	9.38E−92	3.53E−86	0.827252395
ENSG00000132199	E025	ENOSF1	18	−1	5.10E−33	9.59E−28	0.911519852
ENSG00000189283	E004	FHIT	3	−1	1.73E−28	2.18E−23	3.663047029
ENSG00000101019	E025	UQCC1	20	−1	9.24E−28	8.69E−23	0.72317914
ENSG00000111843	E006	TMEM14C	6	1	9.84E−26	7.40E−21	0.91596329
ENSG00000125991	E049	ERGIC3	20	1	9.81E−23	6.15E−18	0.791616483
ENSG00000071082	E025	RPL31	2	1	3.50E−21	1.88E−16	0.717351893
ENSG00000101019	E029	UQCC1	20	−1	2.62E−20	1.23E−15	0.701671242
ENSG00000211644	E016	IGLV1-51	22	1	3.99E−20	1.67E−15	0.441359946
ENSG00000088986	E014	DYNLL1	12	1	5.65E−20	2.12E−15	0.845893878
ENSG00000028310	E077	BRD9	5	−1	3.97E−18	1.36E−13	0.767361674
ENSG00000127586	E055	CHTF18	16	1	2.12E−17	6.64E−13	0.878313474
ENSG00000101019	E007	UQCC1	20	−1	3.20E−17	9.25E−13	1.47227241
ENSG00000160710	E024	ADAR	1	−1	1.30E−16	3.51E−12	1.320985378
ENSG00000101019	E024	UQCC1	20	−1	2.02E−16	5.06E−12	0.71746253
ENSG00000118495	E010	PLAGL1	6	−1	4.10E−16	9.65E−12	2.538692764
ENSG00000255863	E017	AC073610.5	12	−1	1.20E−15	2.65E−11	0.882954589
ENSG00000211644	E017	IGLV1-51	22	1	1.38E−15	2.88E−11	0.485819764
ENSG00000075218	E014	GTSE1	22	1	2.48E−15	4.91E−11	1.468605485
ENSG00000137133	E004	HINT2	9	−1	2.09E−14	3.93E−10	0.839329231
ENSG00000205593	E031	DENND6B	22	−1	5.28E−14	9.46E−10	4.609575952
ENSG00000101019	E013	UQCC1	20	−1	1.05E−13	1.74E−09	2.895712194
ENSG00000101019	E026	UQCC1	20	−1	1.11E−13	1.74E−09	0.732192837
ENSG00000101019	E028	UQCC1	20	−1	1.10E−13	1.74E−09	0.698206407
ENSG00000103426	E028	CORO7-PAM16	16	−1	1.21E−13	1.83E−09	0.838759667
ENSG00000205593	E030	DENND6B	22	−1	3.43E−13	4.97E−09	4.61396886
ENSG00000101019	E010	UQCC1	20	−1	4.54E−13	6.33E−09	1.74387423
ENSG00000111640	E017	GAPDH	12	1	8.37E−13	1.12E−08	1.387332969
ENSG00000100387	E034	RBX1	22	1	1.24E−12	1.61E−08	1.875629945
ENSG00000271270	E006	TMCC1-AS1	3	1	2.12E−12	2.66E−08	2.979442225
ENSG00000028310	E078	BRD9	5	−1	3.00E−12	3.64E−08	0.795339192
ENSG00000101019	E027	UQCC1	20	−1	3.26E−12	3.84E−08	0.703747197
ENSG00000255103	E077	KIAA0754	1	1	3.38E−12	3.85E−08	1.663519412
ENSG00000268400	E035	CTD-3214H19.4	19	1	3.57E−12	3.95E−08	1.463054031
ENSG00000205593	E032	DENND6B	22	−1	4.28E−12	4.60E−08	4.764064117
ENSG00000136044	E042	APPL2	12	−1	5.63E−12	5.88E−08	0.720698938
ENSG00000101019	E005	UQCC1	20	−1	1.00E−11	9.92E−08	1.53637968
ENSG00000167323	E043	STIM1	11	1	9.97E−12	9.92E−08	0.766910234
ENSG00000101019	E016	UQCC1	20	−1	1.35E−11	1.28E−07	2.2692562
ENSG00000161013	E025	MGAT4B	5	−1	1.36E−11	1.28E−07	1.097105348

Genes are ranked by adjusted *P*-value (*P*_adj_).
