# Supplementary material for: Disruption of SF3B1 results in deregulated expression and splicing of key genes and pathways in myelodysplastic syndrome hematopoietic stem and progenitor cells
Source: Leukemia. 2014 Dec 23;29(5):1092–103. doi: 10.1038/leu.2014.331 (PMC4430703; doi:10.1038/leu.2014.331)
Supplement: Supplementary Information [file leu2014331x1.docx]

**Supplementary information**

**Disruption of *SF3B1* results in deregulated expression and splicing of key genes and pathways in myelodysplastic syndrome hematopoietic stem cells**

Hamid Dolatshad^1*^, Andrea Pellagatti^1*^, Marta Fernandez-Mercado^1^, Bon Ham Yip^1^, Luca Malcovati^2^, Martin Attwood^1^, Bartlomiej Przychodzen^3^, Natasha Sahgal^4^, Alexander A. Kanapin^5^, Helen Lockstone^4^, Laura Scifo^1^, Peter Vandenberghe^6^, Elli Papaemmanuil^7^, Chris W.J. Smith^8^, Peter J. Campbell^7^, Seishi Ogawa^9^, Jaroslaw P. Maciejewski^3^, Mario Cazzola^2^, Kienan I. Savage^10^, Jacqueline Boultwood^1^^

**This file contains all legends for the Supplementary Figures and Tables, as well as the Supplementary Materials and Methods.**

**Supplementary Figure S1.** Validation of differentially expressed exons identified by RNA-seq using quantitative real-time PCR.

**Supplementary Figure S2.** *SF3B1* and *ABCB7* expression levels in K562 cells with *SF3B1* knockdown relative to the scramble control, as measured by qRT-PCR up to 10 days post transfection*.*

**Supplementary Figure S3.** Four-set Venn diagram showing the number and overlap of probe sets upregulated (A) or downregulated (B) by >2-fold in K562, TF1, SKM1 and HEL cells treated with *SF3B1* siRNAs compared to cells treated with the scramble control.

**Supplementary Figure S4.** (A) Validation of *SF3B1* (K700E) mutation using Sanger sequencing in one mutant MDS case. (B) Confirmation of *SF3B1* mutations in representative patients by visualization of RNA sequencing reads using IGV software. Two MDS patients with *SF3B1* K700E mutation and variant allele frequency of 45 and 47% are shown alongside a control and a MDS sample with no known splicing gene mutations.

**Supplementary Table S1**. Primers for splicing analysis of *TP53*. Individual bands obtained from PCR-amplified cDNA were gel-extracted and Sanger-sequenced. Sequences were aligned against NM_001126112.

**Supplementary Table S2.** Primers for quantitative splicing analysis of cyclins *CCNA2* and *STK6*.

**Supplementary Table S3.** Clinical characteristics of MDS patients analyzed by RNA sequencing and sequencing metrics for all samples.

**Supplementary Table S4.** Pathways and processes showing coordinated up- or down-regulation identified using Gene Set Enrichment Analysis (GSEA) on expression data from four myeloid cell lines with *SF3B1* knockdown.

**Supplementary Table S5.** Significant differentially expressed genes (fdr<0.05) between *SF3B1* mutant and wildtype, obtained from RNA sequencing data analysis using edgeR. Genes are ranked by adjusted p-value (padj).

**Supplementary Table S6.** Significant differentially expressed genes (fdr<0.05) between *SF3B1* mutant and control, obtained from RNA sequencing data analysis using edgeR. Genes are ranked by adjusted p-value (padj).

**Supplementary Table S7.** Pathway analysis (IPA) of the significant differentially expressed genes between *SF3B1* mutant and control obtained using edgeR.

**Supplementary Table S8.** Gene Set Enrichment Analysis (GSEA) of RNA-sequencing expression data of *SF3B1* mutant versus control and of *SF3B1* mutant versus wildtype. Enriched gene sets showing upregulation are highlighted in green and those showing downregulation are highlighted in red.

**Supplementary Table S9.** Differential exon usage (fdr<0.05) between *SF3B1* mutant and control, obtained from RNA sequencing data analysis using DEXSeq. ExonIDs are ranked by adjusted p-value (padj).

**Supplementary Table S10.** Differential exon usage (fdr<0.05) between *SF3B1* mutant and wildtype, obtained from RNA sequencing data analysis using DEXSeq. ExonIDs are ranked by adjusted p-value (padj).

**Supplementary Table S11.**Pathway analysis (IPA) of the genes showing significant differential exon usage between *SF3B1* mutant and wildtype obtained using DEXSeq .

**Supplementary Table S12.** Pathway analysis (IPA) of the genes showing significant differential exon usage between *SF3B1* mutant and control obtained using DEXSeq.

**Supplementary Table S13.** Overlap between the list of genes bound by the BRCA1-BCLAF1-SF3B1 complex and the significant genes identified by edgeR and DEXSeq. Genes highlighted in green are common in both comparisons of *SF3B1* mutant to wildtype and to control.

**Supplementary Materials and Methods**

*SF3B1 mutation screening in cell lines*

The four myeloid cell lines (TF1, K562, HEL and SKM1) were screened for *SF3B1* mutations within mutation hotspots by Sanger sequencing using the conditions described in the following table.

| Exon | Forward primer | Reverse primer | Annealing temp | Elongation period | PCR cycles |
| --- | --- | --- | --- | --- | --- |
| 12 | 5’-TGGAAATGAACTCATGCTGTC-3’ | 5’-TGCAAAGGAAAAGGTCTAGGA-3’ | 59 C | 1min | 35 |
| 13-14 | 5’-TTCTGTACATGAGCATTTCATCA-3’ | 5’-GACAGGCTGTGTGTCTACCTCT-3’ | 59 C | 1min | 35 |
| 15-16 | 5’-CTGCAGTTTGGCYGAATAGTTG-3’ | 5’-AAAATTCTGTTAGAACCATGAAACA-3’ | 59 C | 1min | 35 |

*Validation of differentially expressed exons by quantitative real-time PCR*

Total RNA from bone marrow CD34^+^ cells of the eight *SF3B1* mutant, four wildtype and five control used for RNA-seq experiments was reverse-transcribed using Retroscript kit (Ambion, Life Technologies, UK). The expression levels of exons within the *AKAP9*, *EFCAB13* (differentially expressed between *SF3B1* mutant and control) and *FSIP2* (differentially expressed between *SF3B1* mutant and wildtype) genes were determined using custom TaqMan expression assays (Applied Biosystems, Foster City, CA, USA) specific for the affected exon. *B2M* expression levels were used to normalize for differences in input cDNA. Samples were run on a LightCycler 96 Real-time PCR system (Roche Diagnostics, Lewes, UK) and expression ratios were calculated using the ddCT method.
