# Supplementary material for: Disruption of SF3B1 results in deregulated expression and splicing of key genes and pathways in myelodysplastic syndrome hematopoietic stem and progenitor cells
Source: Leukemia. 2014 Dec 23;29(5):1092–103. doi: 10.1038/leu.2014.331 (PMC4430703; doi:10.1038/leu.2014.331)
Supplement: Supplementary Figures S1-4 [file leu2014331x2.ppt]

## Slide 1
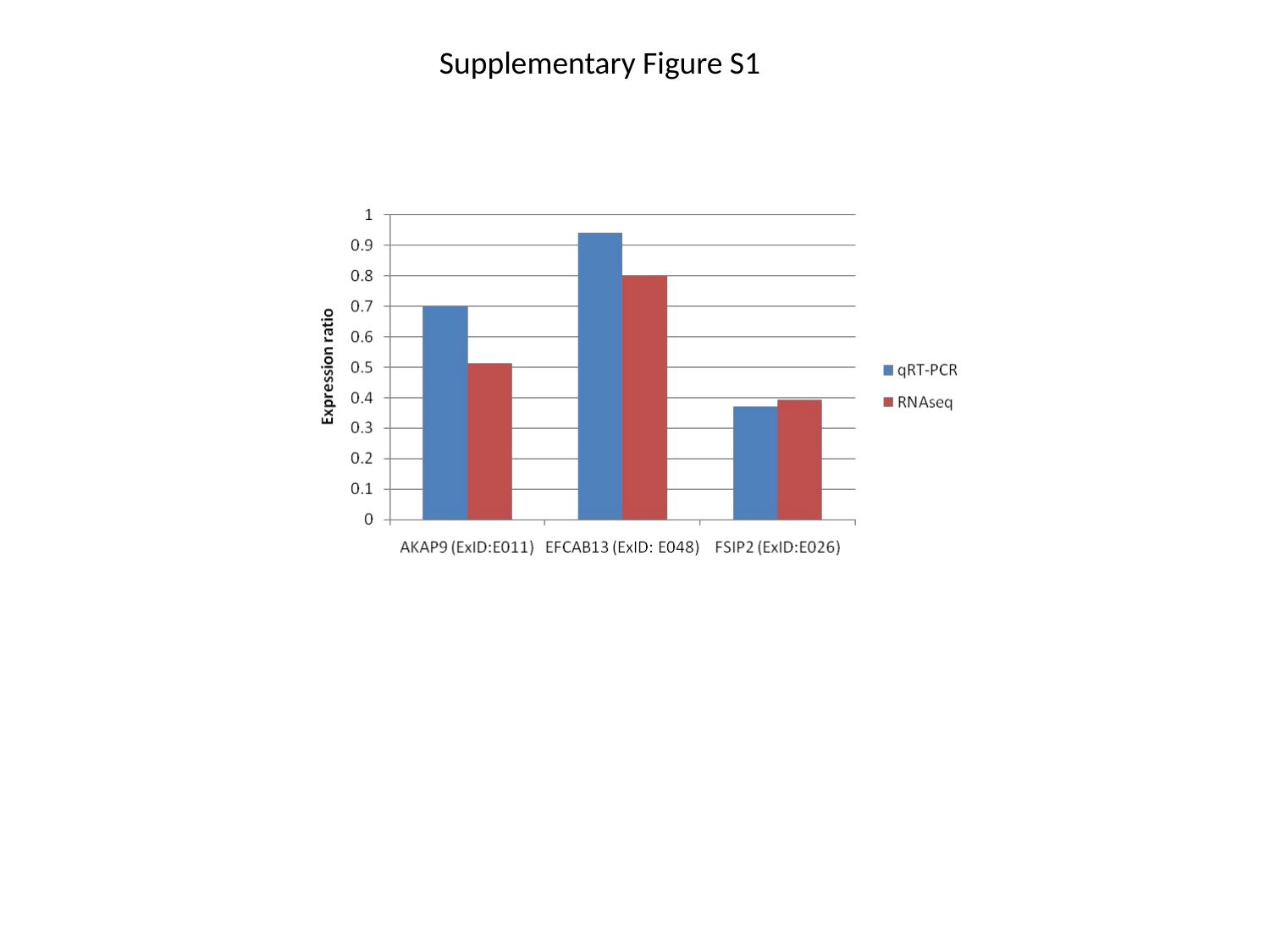

Supplementary Figure S1

## Slide 2
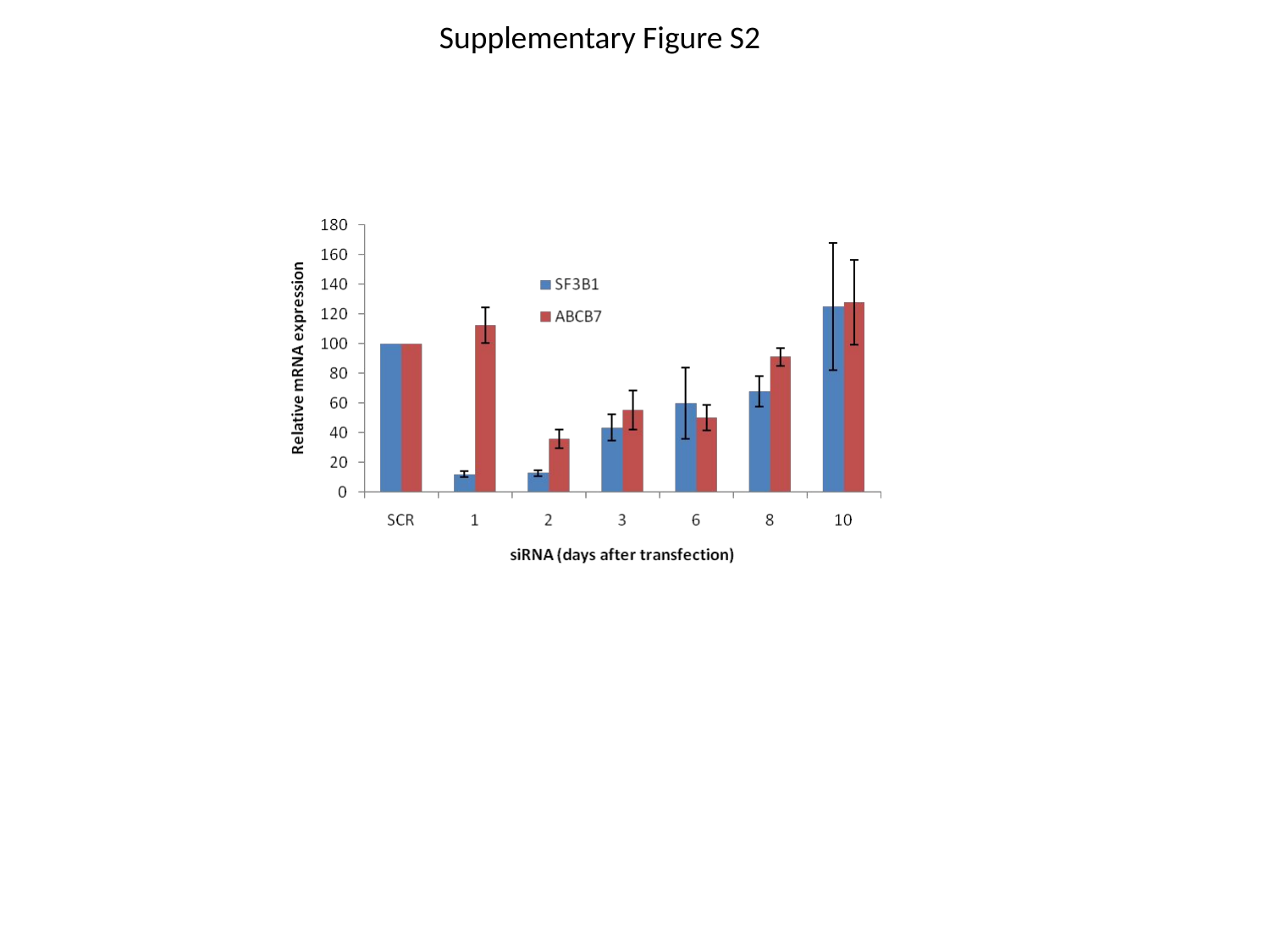

Supplementary Figure S2

## Slide 3
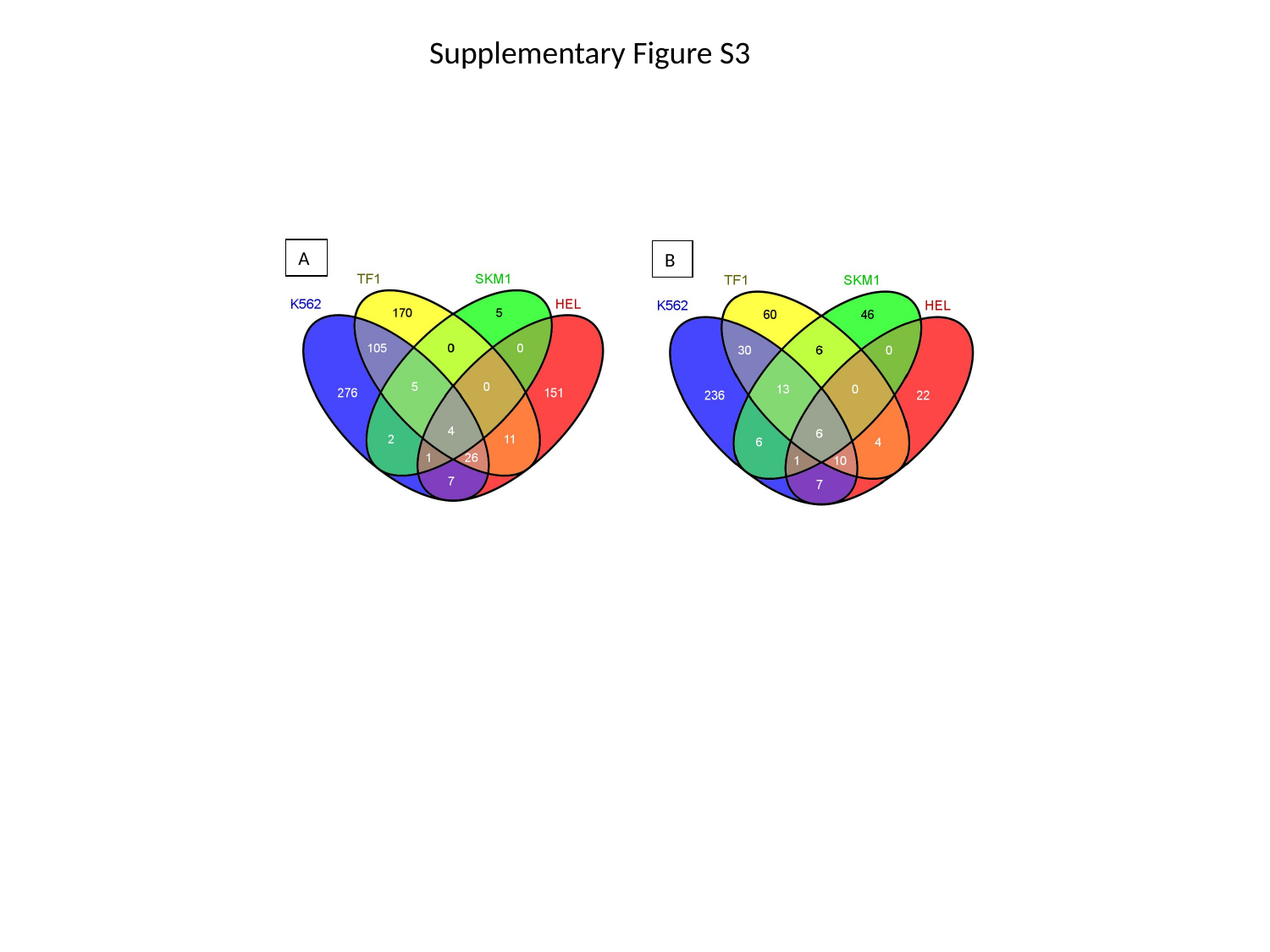

Supplementary Figure S3
A
B

## Slide 4
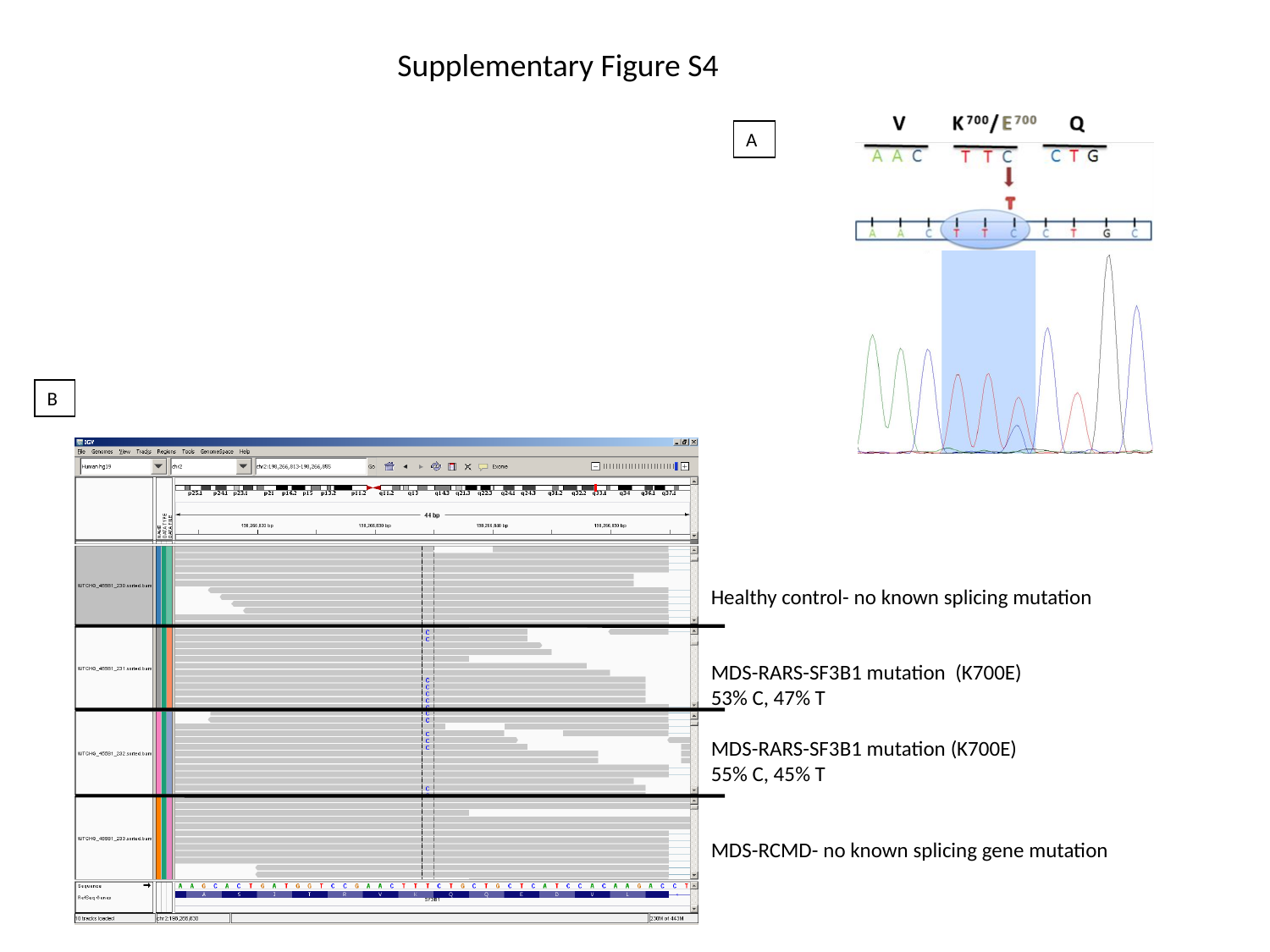

Supplementary Figure S4
A
B
Healthy control- no known splicing mutation
MDS-RARS-SF3B1 mutation (K700E)
53% C, 47% T
MDS-RARS-SF3B1 mutation (K700E)
55% C, 45% T
MDS-RCMD- no known splicing gene mutation
