# Supplementary material for: Disruption of SF3B1 results in deregulated expression and splicing of key genes and pathways in myelodysplastic syndrome hematopoietic stem and progenitor cells
Source: Leukemia. 2014 Dec 23;29(5):1092–103. doi: 10.1038/leu.2014.331 (PMC4430703; doi:10.1038/leu.2014.331)
Supplement: Supplementary Table 1 [file leu2014331x3.docx]

| **Primer ID** | **Primer seq** | **target** | **Splicing analysis** |
| --- | --- | --- | --- |
| TP53_P1_SPL_EX1F | GTGACACGCTTCCCTGGATT | Exons 1-11 | Many alternate isoforms. Refer to Wei *et al*., J Nucleic Acids, 2012; 2012:687359 |
| TP53_SPL_EX11R | TCAGGCCCTTCTGTCTTGAA |  |  |
| TP53_P2_SPL_EX5F | TACTCCCCTGCCCTCAACAA | Exons 5-11 |  |
| TP53_SPL_EX11R | TCAGGCCCTTCTGTCTTGAA |  |  |
