# Supplementary material for: Disruption of SF3B1 results in deregulated expression and splicing of key genes and pathways in myelodysplastic syndrome hematopoietic stem and progenitor cells
Source: Leukemia. 2014 Dec 23;29(5):1092–103. doi: 10.1038/leu.2014.331 (PMC4430703; doi:10.1038/leu.2014.331)
Supplement: Supplementary Table 2 [file leu2014331x4.docx]

| **Target gene** | **Event detected** | **Primer pair** |
| --- | --- | --- |
| *CCNA2* (cyclin A2) | Skipping of exon 5 | CCNA2_e4c_F GCTATCCTCGTGGACTGGTT  CCNA2_e4c_R CCTCTCAGCACTGACATGGA |
|  |  | CCNA2_e4_e6_F TGCTATGCTGTTAGCCTCTTTTT  CCNA2_e6_R CCCGTGACTGTGTAGAGTGC |
|  |  | CCNA2_e4_e5_C_F TGCTATGCTGTTAGCCTCAAA  CCNA2_e5_R AGGCTGCTGATGCAGAAAGT |
|  |  | CCNA2_e5_e6_F TTGAAAGTTTAGCAATGTTTTTGG  CCNA2_e6_R AAAGGCAGCTCCAGCAATAA |
|  |  | CCNA2_e5_B_F2 CCCAGAAGTAGCGGAGTTTG  CCNA2_e5_B_R AGGCTGCTGATGCAGAAAGT |
| *STK6* (AURKA) | Skipping of exons 4, 5 and 6 | STK6_e3c_F AATTCTTCCCAGCGCATTC  STK6_e3c_R AGGGGCTGCTTGCTCTTT |
|  |  | STK6_e3c_F AATTCTTCCCAGCGCATTC  STK6_e3c_e4_R TCCTCAGGATTATTTTCAGGTG |
|  |  | STK6_e3_e7_F CTGCCATCGGCACCTGTATAT  STK6_e7_R GCATGTACTGACCACCCAAA |
